# Supplementary figures and images for: Pch2 orchestrates the meiotic recombination checkpoint from the cytoplasm
Source: PLoS Genet. 2021 Jul 14;17(7):e1009560. doi: 10.1371/journal.pgen.1009560 (PMC8312941; doi:10.1371/journal.pgen.1009560)

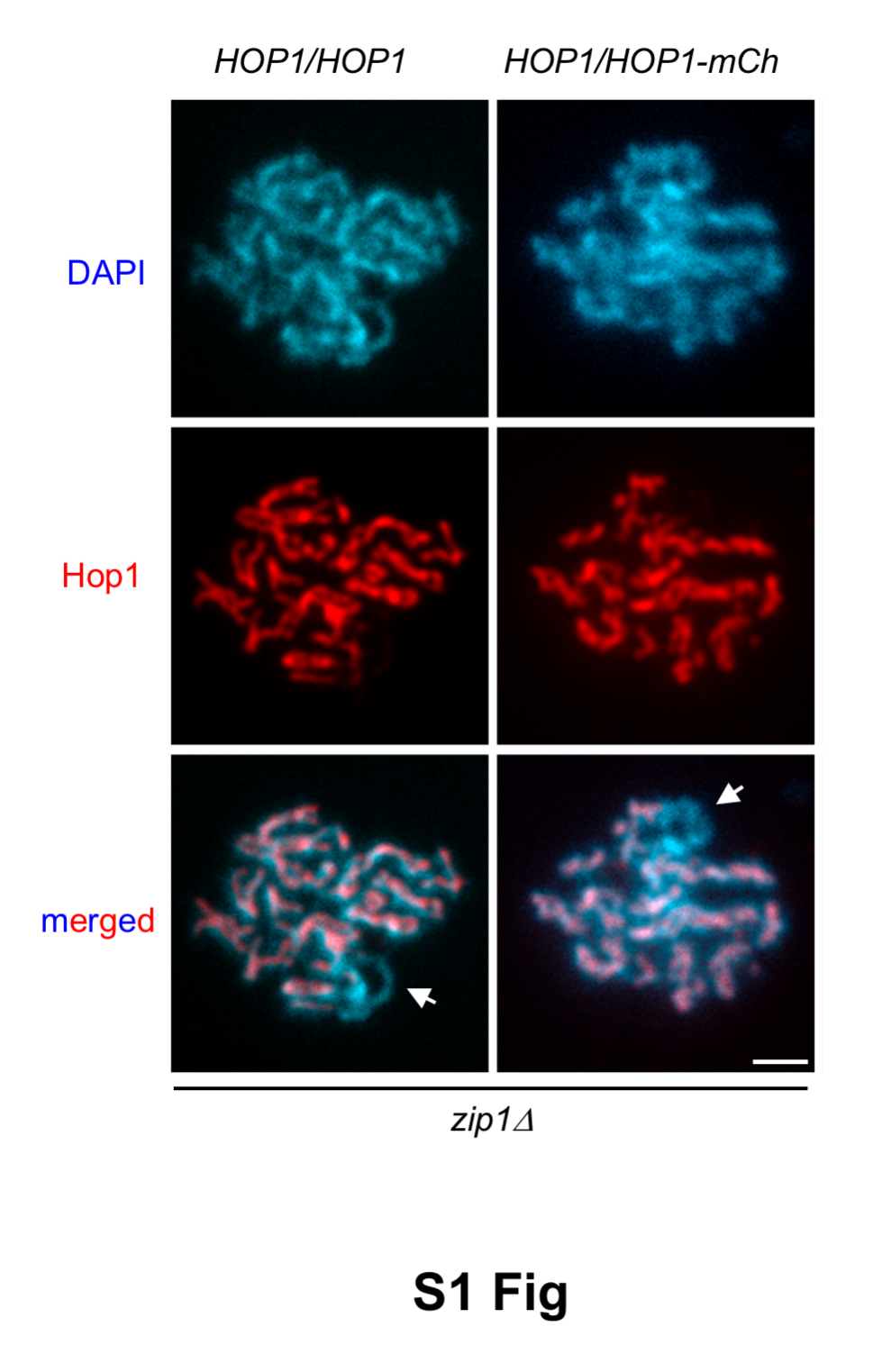

Supplement: S1 Fig — Immunofluorescence of meiotic chromosomes stained with anti-Hop1 (red) and DAPI (blue). Arrows point to the rDNA region devoid of Hop1. Spreads were prepared at 16 h. Scale bar, 2 μm. Strains are: DP422 (zip1Δ HOP1/HOP1) and DP1500 (zip1Δ HOP1/HOP1-mCherry). (TIF) [file pgen.1009560.s001.tif]

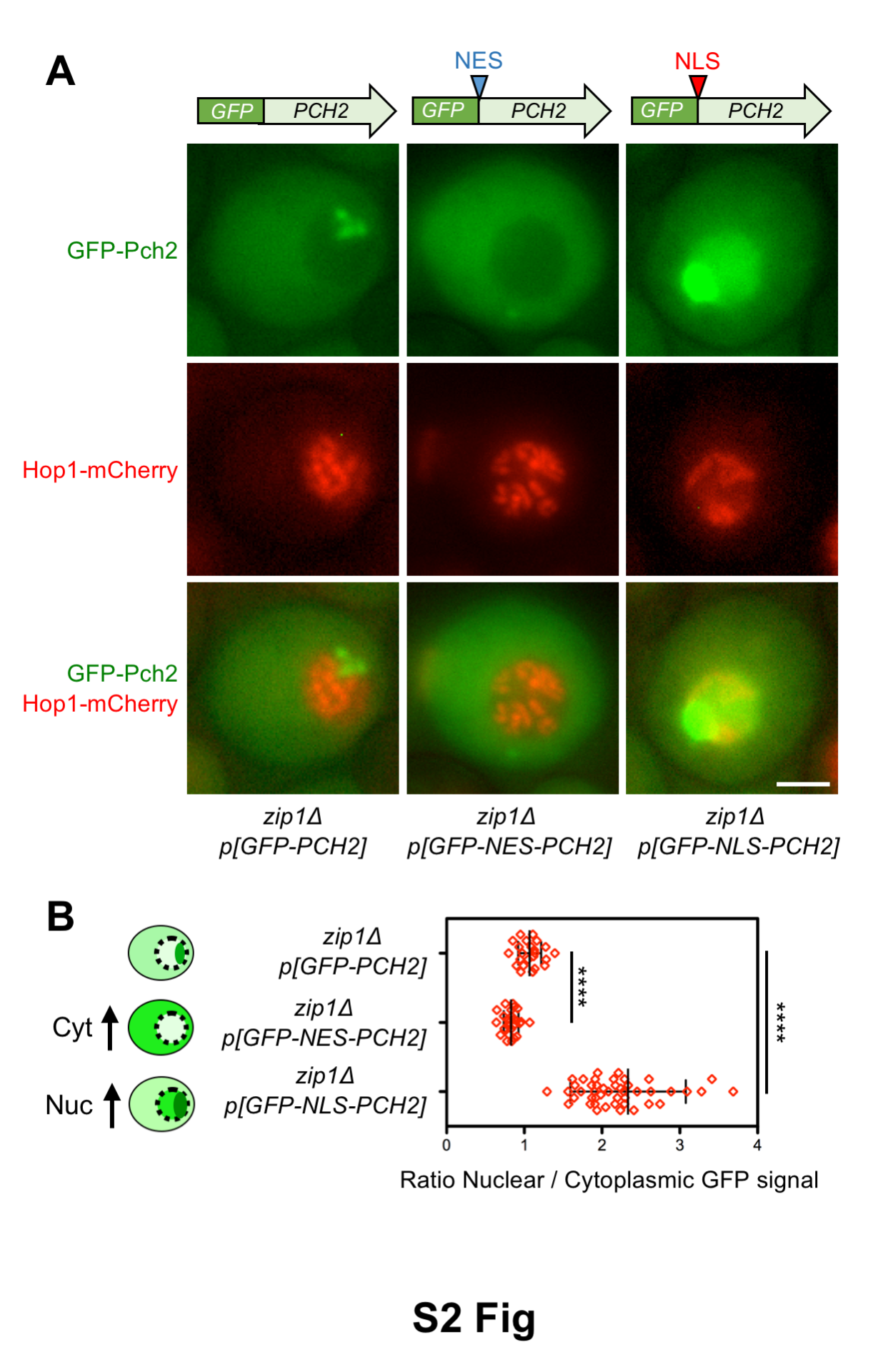

Supplement: S2 Fig — (A) Fluorescence microscopy analysis of plasmid-expressed GFP-Pch2, GFP-NES-Pch2 or GFP-NLS-Pch2 (green) and Hop1-mCherry (red) in whole meiotic cells 15 h after meiotic induction. Representative cells are shown. Scale bar, 2 μm. (B) Quantification of the ratio of nuclear (including nucleolar) to cytoplasmic GFP fluorescent signal. Error bars: SD. The cartoon illustrates the subcellular localization of the different versions of GFP-Pch2 (green). The strain in (A) and (B) is DP1500 (zip1Δ) transformed with the centromeric plasmids pSS393 (GFP-PCH2), pSS408 (GFP-NES-PCH2) and pSS421 (GFP-NLS-PCH2). (TIF) [file pgen.1009560.s002.tif]

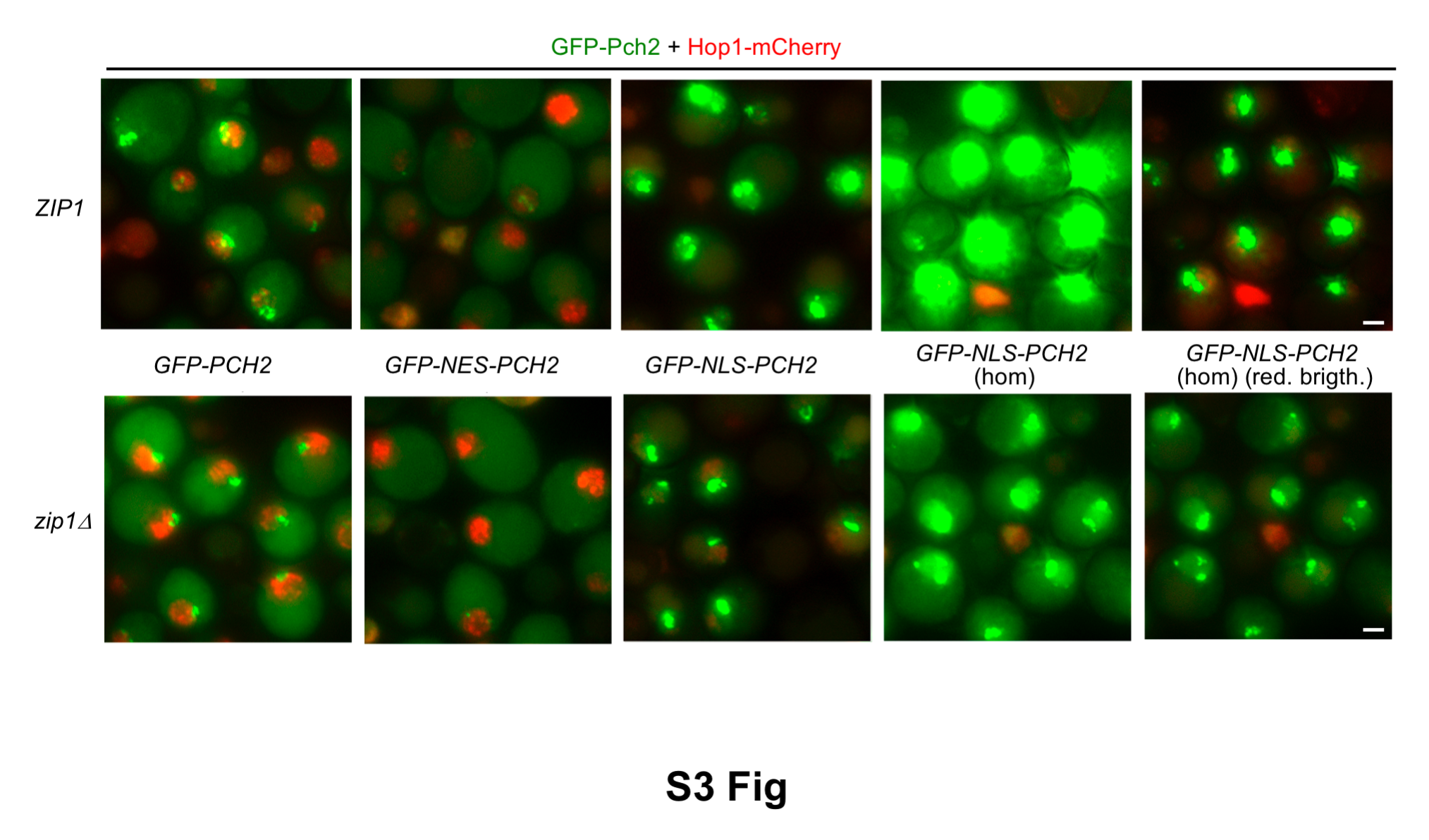

Supplement: S3 Fig — Additional representative fields corresponding to the fluorescence microscopy analysis of localization of GFP-Pch2, GFP-NES-Pch2 or GFP-NLS-Pch2 (green) and Hop1-mCherry (red) presented in Fig 3. Scale bar, 2 μm. (TIF) [file pgen.1009560.s003.tif]

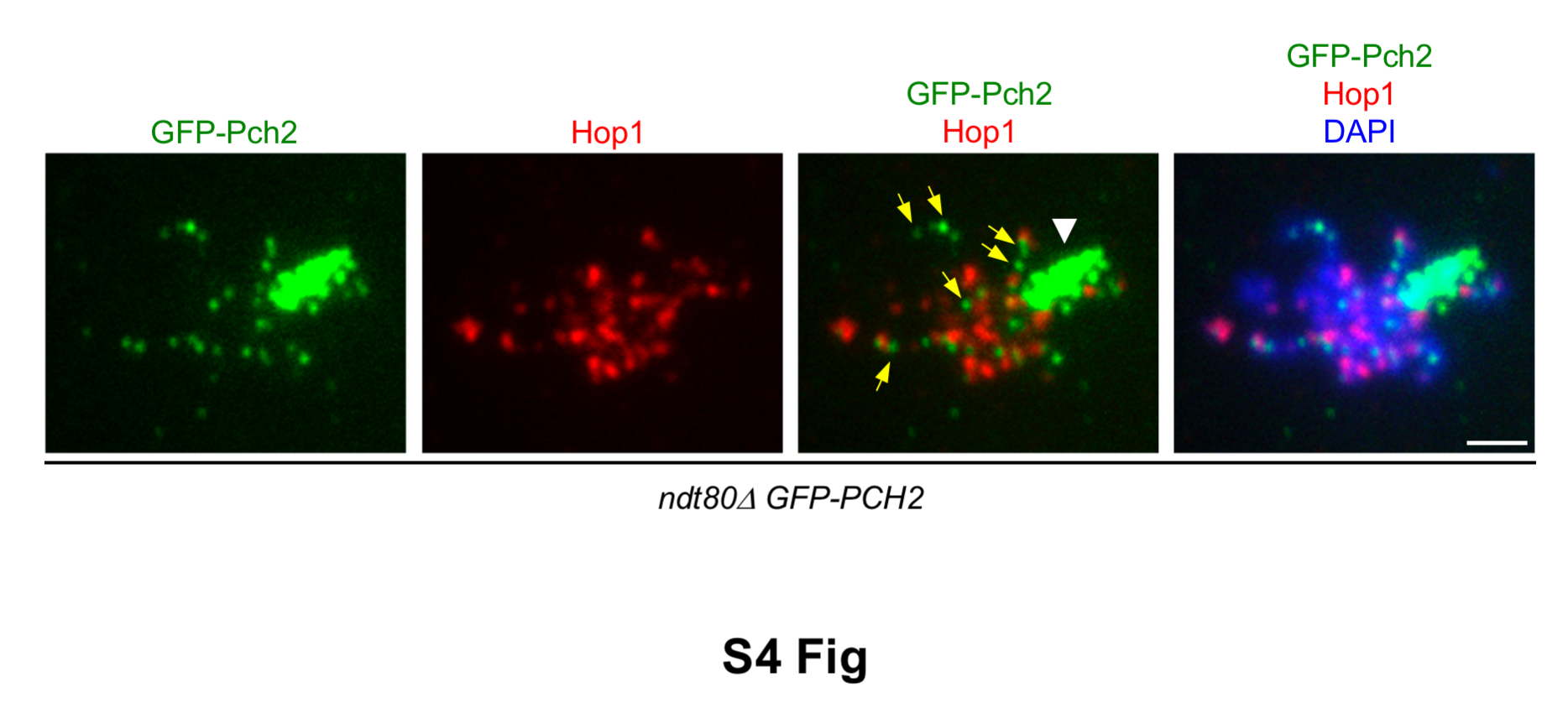

Supplement: S4 Fig — Immunofluorescence of meiotic chromosomes stained with anti-GFP antibodies (to detect GFP-Pch2; green), anti-Hop1 antibodies (red) and DAPI (blue). White arrowhead points to the rDNA. Yellow arrows point to interstitial GFP-Pch2 foci alternating with Hop1 signal. The Pch2 signal was computer-enhanced to visualize chromosomal foci. Spreads were prepared from ndt80Δ strains at 24 h. Scale bar, 2 μm. The strain is DP1654. (TIF) [file pgen.1009560.s004.tif]

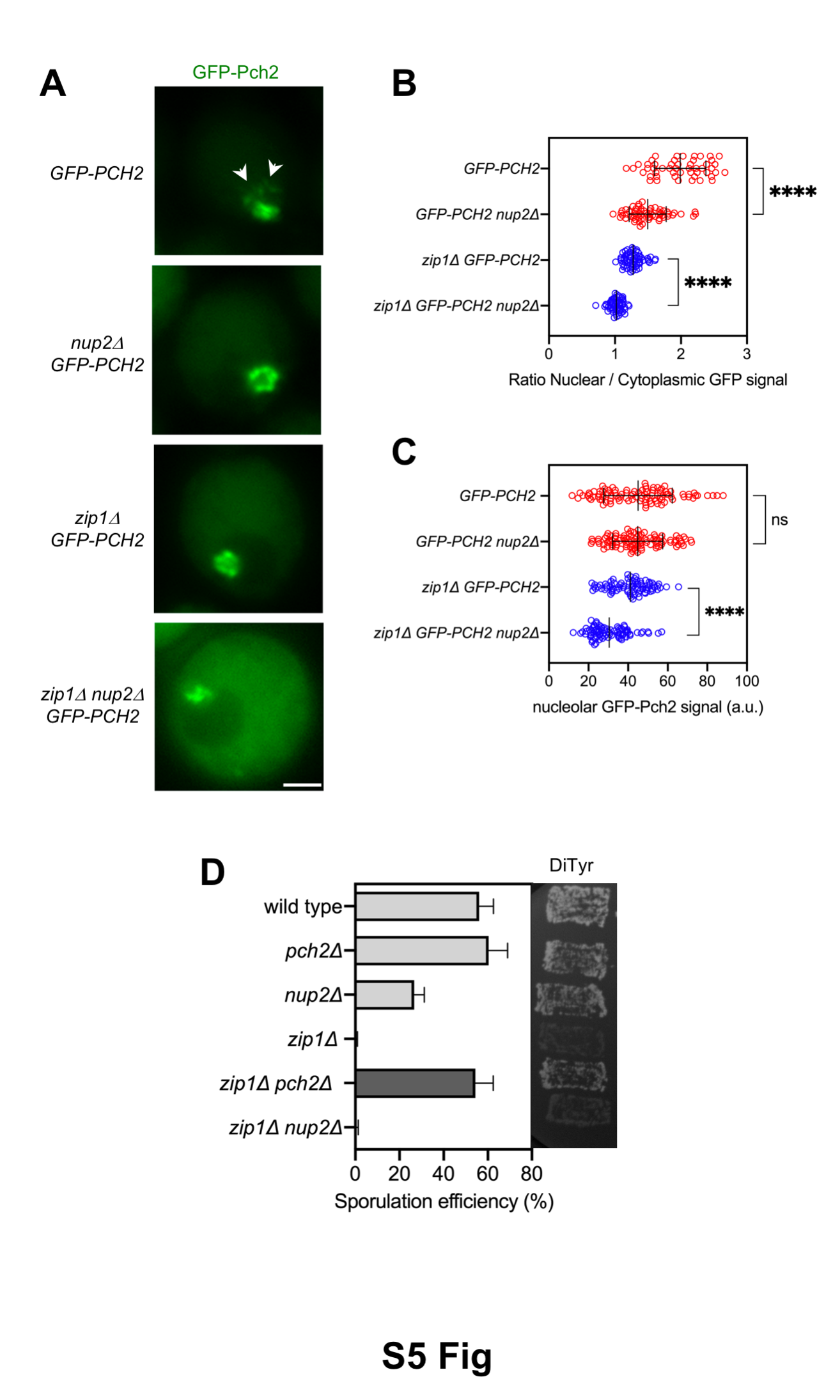

Supplement: S5 Fig — (A) Fluorescence microscopy analysis of GFP-Pch2 distribution in whole meiotic cells of the indicated genotypes 16 hours after meiotic induction. Representative cells are shown. Arrows point to chromosomal (non-nucleolar) Pch2. Scale bar, 2 μm. (B, C) Quantification of the ratio of nuclear (including nucleolar) to cytoplasmic mean GFP fluorescent signal (B) and the nucleolar GFP-Pch2 signal (C) in cells analyzed as in (A). Error bars: SD. (D) Sporulation efficiency, assessed by microscopic counting of asci, and dityrosine fluorescence (DiTyr), as a visual indicator of sporulation, were examined after 3 days on sporulation plates. Error bars, SD; n = 3. At least 300 cells were counted for each strain. Strains in (A-C) are: DP1624 (GFP-PCH2), DP1744 (nup2Δ GFP-PCH2), DP1625 (zip1Δ GFP-PCH2) and DP1745 (zip1Δ nup2Δ GFP-PCH2). Strains in (D) are: DP1151 (wild type), DP1164 (pch2Δ), DP1723 (nup2Δ), DP1152 (zip1Δ), DP1161(zip1Δ pch2Δ) and DP1724 (zip1Δ nup2Δ). (TIF) [file pgen.1009560.s005.tif]

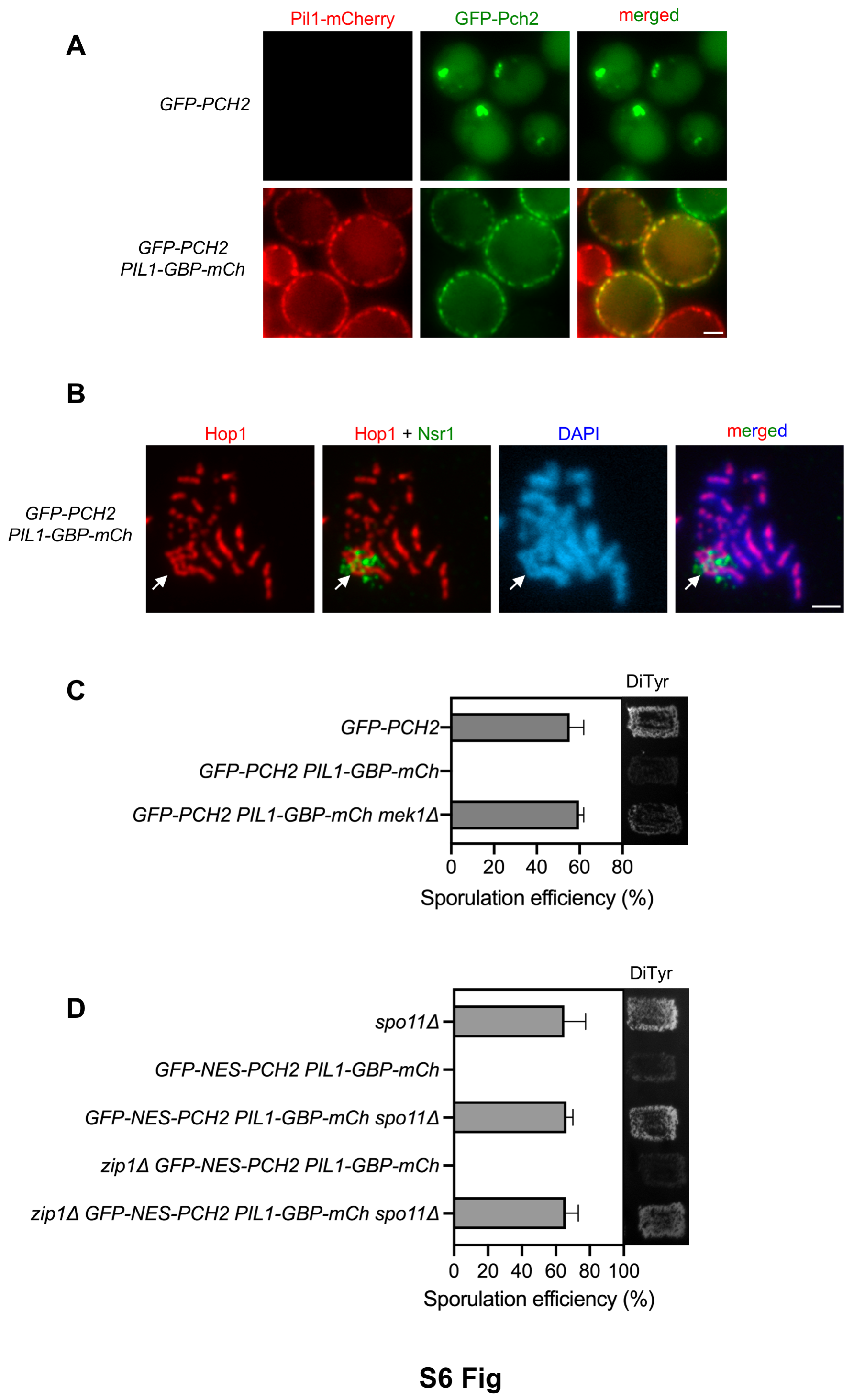

Supplement: S6 Fig — (A) Fluorescence microscopy analysis of GFP-Pch2 and Pil1-GBP-mCherry distribution in whole meiotic cells of the indicated genotypes 16 hours after meiotic induction. Scale bar, 2 μm. (B) Immunofluorescence of meiotic chromosomes stained with anti-Hop1 (red) and anti-Nsr1 (nucleolar marker; green) antibodies, and DAPI (blue). The arrow points to the rDNA region. Spreads were prepared at 16 h. Scale bar, 2 μm. (C) Sporulation efficiency and dityrosine fluorescence (DiTyr), were examined after 3 days on sporulation plates. Error bars, SD; n = 3. At least 300 cells were counted for each strain. Strains in (A-C) are: DP1624 (GFP-PCH2), DP1797 (GFP-PCH2 PIL1-GBP-mCherry) and DP1813 (GFP-PCH2 PIL1-GBP-mCherry mek1Δ). (D) Deletion of SPO11 alleviates the sporulation block resulting from GFP-NES-Pch2 tethering to the plasma membrane. Sporulation efficiency and dityrosine fluorescence (DiTyr), were analyzed as in (C). Error bars, SD; n = 3. Strains in (D) are: DP1523 (spo11Δ), DP1795 (GFP-NES-PCH2 PIL1-GBP-mCherry), DP1846 (GFP-NES-PCH2 PIL1-GBP-mCherry spo11Δ), DP1796 (zip1Δ GFP-NES-PCH2 PIL1-GBP-mCherry) and DP1847 (zip1Δ GFP-NES-PCH2 PIL1-GBP-mCherry spo11Δ). (TIF) [file pgen.1009560.s006.tif]

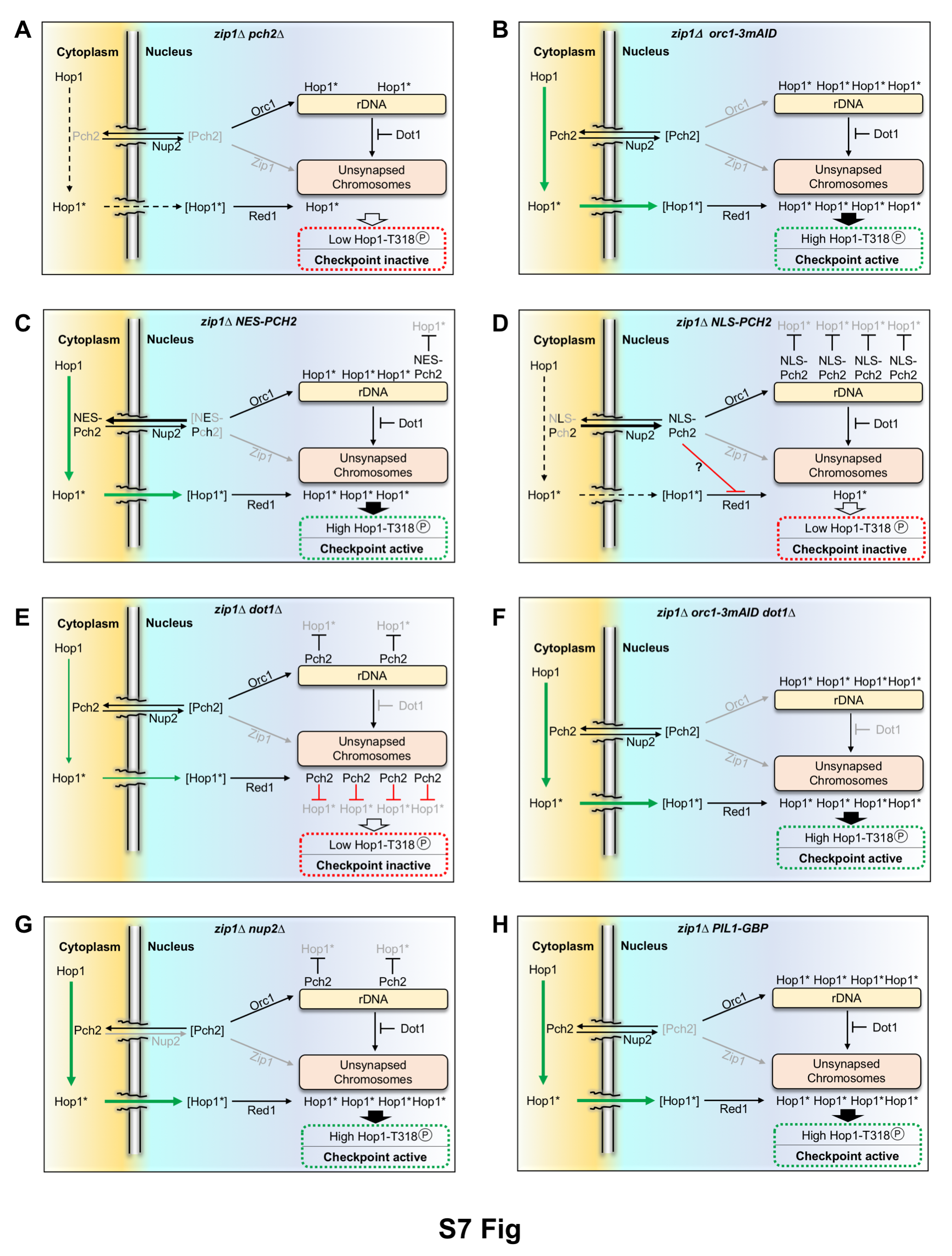

Supplement: S7 Fig — (A) In zip1Δ pch2Δ, the conformational change required for Hop1 chromosomal incorporation is inefficient leading to low levels of Hop1-T318 phosphorylation and checkpoint defects. (B) In zip1Δ orc1-3mAID, Pch2 is not recruited to the rDNA resulting in its accumulation in the cytoplasm fostering proficient Hop1 loading and activation. (C) In zip1Δ NES-PCH2, the balance of Pch2 distribution is biased to the cytoplasm also supporting checkpoint activation. (D) In zip1Δ NLS-PCH2, the balance of Pch2 distribution is skewed towards the nucleus resulting in the accumulation of the protein in the rDNA and the nucleoplasm. The checkpoint defect in zip1Δ NLS-PCH2 likely stems from the reduced levels of cytoplasmic Pch2. However, since increased dosage of NLS-Pch2 causes a stronger checkpoint defect, it is possible that the accumulation of NLS-Pch2 in the nucleoplasm also exerts an inhibitory effect on checkpoint activity. (E) In zip1Δ dot1Δ, Pch2 loses its rDNA confinement and it is widely distributed throughout unsynapsed chromosomes provoking Hop1 release and, therefore, low levels of Hop1-T318 phosphorylation. (F) In zip1Δ orc1-3mAID dot1Δ, the inability of Pch2 to be recruited to the rDNA results in its exclusive cytoplasmic localization supporting checkpoint activation. Since in the absence of Orc1 there is no Pch2 to be confined in the rDNA, Dot1 is irrelevant in this context. (G) In zip1Δ nup2Δ, the pool of cytoplasmic Pch2 is increased likely reflecting a defect in Pch2 import to the nucleus in the absence of the nucleoporin; consequently, the amount of nucleolar Pch2 is reduced. The presence of Pch2 in the cytoplasm ensures an efficient checkpoint response. (H) In zip1Δ PIL1-GBP, the GFP-tagged Pch2 is sequestered in the eisosomes facing the cytoplasmic side of the plasma membrane and, therefore, being proficient in the generation of the Hop1 conformational state that facilitates chromosome incorporation. Furthermore, since in this situation Pch2 is tightly trapped o [file pgen.1009560.s007.tif]

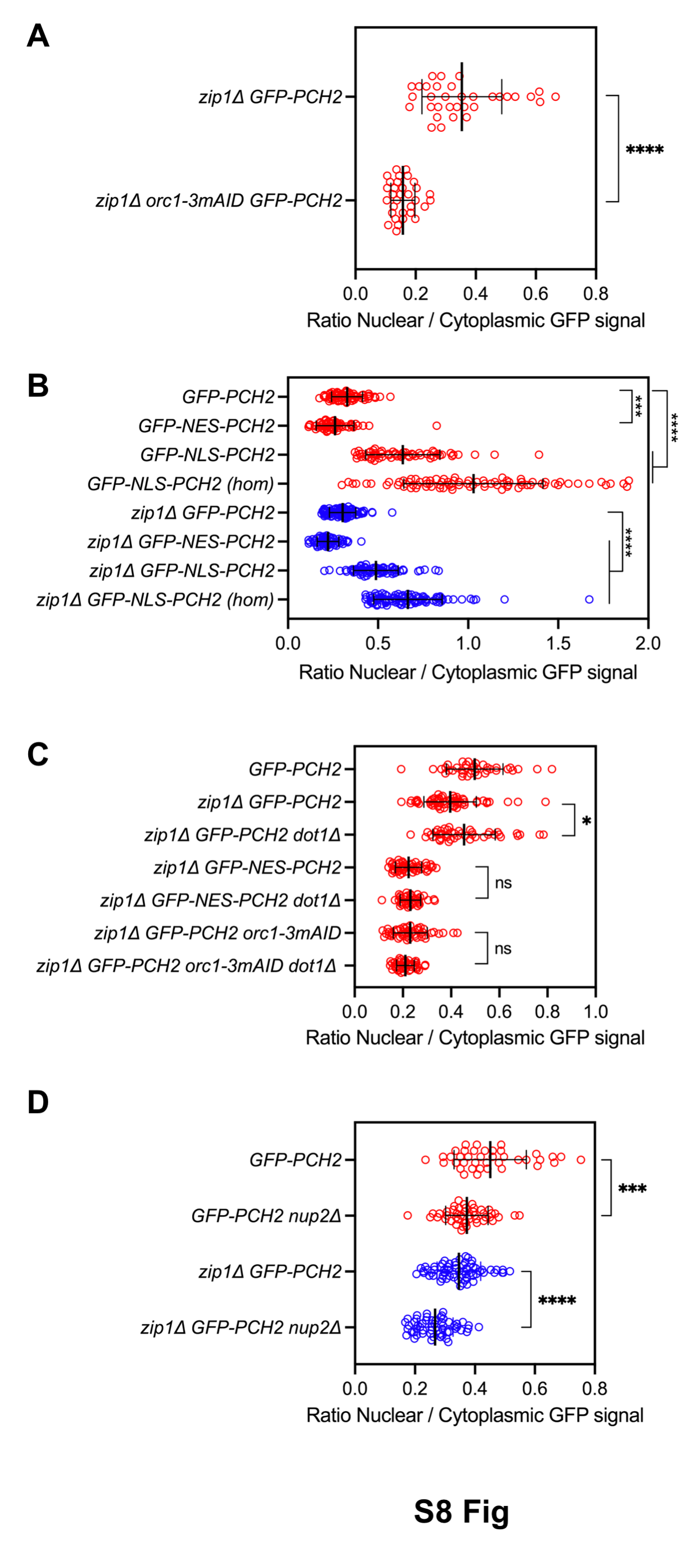

Supplement: S8 Fig — Quantification of the ratio of nuclear (including nucleolar) to cytoplasmic GFP fluorescent signal using total intensity values (integrated density) within the contour of the nuclear and cytoplasmic area selected. Error bars: SD. (A) Measurements corresponding to the experiments presented in Fig 1C and 1D. (B) Measurements corresponding to the experiments presented in Fig 3A and 3B. (C) Measurements corresponding to the experiments presented in Fig 6A and 6B. (D) Measurements corresponding to the experiments presented in S5A and S5B Fig. (TIF) [file pgen.1009560.s008.tif]
