# Supplementary material for: Pch2 orchestrates the meiotic recombination checkpoint from the cytoplasm
Source: PLoS Genet. 2021 Jul 14;17(7):e1009560. doi: 10.1371/journal.pgen.1009560 (PMC8312941; doi:10.1371/journal.pgen.1009560)
Supplement: S1 Table — (PDF) [file pgen.1009560.s009.pdf]

S1 Table. ***Saccharomyces cerevisiae*** **strains**

Strain	Genotype*	Source
BR1919-2N	*MAT*a/*MAT*α *leu2-3,112 his4-260 thr1-4 trp1-289 ura3-1 ade2-1*	Roeder Lab
DP421	BR1919-2N *lys2ΔNheI*	PSS Lab
DP422	DP421 *zip1Δ::LYS2*	PSS Lab
DP424	DP421 *ndt80Δ::LEU2*	PSS Lab
DP428	DP421 *zip1Δ::LYS2 ndt80Δ::LEU2*	PSS Lab
DP881	DP421 *zip1Δ::LYS2 pch2Δ::TRP1 ndt80Δ::LEU2*	PSS Lab
DP1023	DP421 *pch2Δ::TRP1*	PSS Lab
DP1029	DP421 *zip1Δ::LYS2 pch2Δ::TRP1*	PSS Lab
DP1058	DP421 *pch2Δ::TRP1 ndt80Δ::LEU2*	PSS Lab
DP1151	BR1919-2N *3HA-PCH2*	PSS Lab
DP1152	BR1919-2N *zip1Δ::LEU2 3HA-PCH2*	PSS Lab
DP1161	BR1919-2N *zip1Δ::LEU2 pch2Δ::TRP1*	PSS Lab
DP1164	BR1919-2N *pch2Δ::TRP1*	PSS Lab
DP1405	DP421 *zip1Δ::LEU2 pch2Δ::URA3*	PSS Lab
DP1500	DP421 *zip1Δ::LYS2 HOP1-mCherry::natMX4/HOP1*	PSS Lab
DP1523	DP421 *spo11Δ::ADE2*	PSS Lab
DP1620	BR1919-2N P_{HOP1}*-GFP-PCH2*	This work
DP1621	BR1919-2N *zip1Δ::LEU2* P_{HOP1}*-GFP-PCH2*	This work
DP1624	BR1919-2N P_{HOP1}*-GFP-PCH2/pch2Δ::TRP1*	This work
DP1625	BR1919-2N *zip1Δ::LEU2* P_{HOP1}*-GFP-PCH2/pch2Δ::TRP1*	This work
DP1630	BR1919-2N *zip1Δ::LEU2* P_{HOP1}*-GFP-PCH2 orc1-3mAID::hphNT* P_{HOP1}*-OsTIR1::URA3 ndt80Δ::kanMX3*	This work
DP1633	BR1919-2N *zip1Δ::LEU2* P_{HOP1}*-GFP-PCH2 orc1-3mAID::hphNT* P_{HOP1}*-OsTIR1::URA3 HOP1-mCherry::natMX4/HOP1*	This work
DP1636	BR1919-2N *zip1Δ::LEU2* P_{HOP1}*-GFP-PCH2 HOP1-mCherry::natMX4/HOP1*	This work
DP1640	BR1919-2N *zip1Δ::LEU2* P_{HOP1}*-GFP-PCH2 ndt80Δ::kanMX3 lys2/LYS2*	This work
DP1644	BR1919-2N *zip1Δ::LEU2* P_{HOP1}*-GFP-PCH2/pch2Δ::TRP1 orc1-3mAID::hphNT* P_{HOP1}*-OsTIR1::URA3*	This work
DP1650	BR1919-2N P_{HOP1}*-GFP-PCH2/pch2Δ::TRP1 HOP1-mCherry::natMX4/HOP1*	This work

DP1651	BR1919-2N *zip1Δ::LEU2* P_{HOP1}*-GFP-PCH2/pch2Δ::TRP1 HOP1-mCherry::natMX4/HOP1*	This work
DP1654	BR1919-2N P_{HOP1}*-GFP-PCH2/pch2Δ::TRP1 ndt80Δ::kanMX3*	This work
DP1655	BR1919-2N *zip1Δ::LEU2* P_{HOP1}*-GFP-PCH2/pch2Δ::TRP1 ndt80Δ::kanMX3 lys2/LYS2*	This work
DP1669	DP421 P_{HOP1}*-GFP-NES-PCH2*	This work
DP1670	DP421 *zip1Δ::LYS2* P_{HOP1}*-GFP-NES-PCH2*	This work
DP1685	DP421 P_{HOP1}*-GFP-NES-PCH2/pch2Δ::TRP1*	This work
DP1686	DP421 *zip1Δ::LYS2* P_{HOP1}*-GFP-NES-PCH2/pch2Δ::TRP1*	This work
DP1687	DP421 P_{HOP1}*-GFP-NES-PCH2/pch2Δ::TRP1 HOP1-mCherry::natMX4/HOP1*	This work
DP1688	DP421 *zip1Δ::LYS2* P_{HOP1}*-GFP-NES-PCH2/pch2Δ::TRP1 HOP1-mCherry::natMX4/HOP1*	This work
DP1695	DP421 P_{HOP1}*-GFP-NLS-PCH2*	This work
DP1696	DP421 *zip1Δ::LYS2* P_{HOP1}*-GFP-NLS-PCH2*	This work
DP1697	DP421 P_{HOP1}*-GFP-NLS-PCH2 HOP1-mCherry::natMX4/HOP1*	This work
DP1698	DP421 *zip1Δ::LYS2* P_{HOP1}*-GFP-NLS-PCH2 HOP1-mCherry::natMX4/HOP1*	This work
DP1699	DP421 P_{HOP1}*-GFP-NLS-PCH2/pch2Δ::TRP1*	This work
DP1700	DP421 P_{HOP1}*-GFP-NLS-PCH2/pch2Δ::TRP1 HOP1-mCherry::natMX4/HOP1*	This work
DP1701	DP421 *zip1Δ::LYS2* P_{HOP1}*-GFP-NLS-PCH2/pch2Δ::TRP1*	This work
DP1702	DP421 *zip1Δ::LYS2* P_{HOP1}*-GFP-NLS-PCH2/pch2Δ::TRP1 HOP1-mCherry::natMX4/HOP1*	This work
DP1723	BR1919-2N *3HA-PCH2 nup2Δ::hphMX4*	This work
DP1724	BR1919-2N *zip1Δ::LEU2 3HA-PCH2 nup2Δ::hphMX4*	This work
DP1725	DP421 P_{HOP1}*-GFP-NES-PCH2/pch2Δ::TRP1 ndt80Δ::kanMX6*	This work
DP1726	DP421 *zip1Δ::LYS2* P_{HOP1}*-GFP-NES-PCH2/pch2Δ::TRP1 ndt80Δ::kanMX6*	This work
DP1729	DP421 P_{HOP1}*-GFP-NLS-PCH2/pch2Δ::TRP1 ndt80Δ::kanMX6*	This work
DP1730	DP421 *zip1Δ::LYS2* P_{HOP1}*-GFP-NLS-PCH2/pch2Δ::TRP1 ndt80Δ::kanMX6*	This work
DP1734	DP421 *zip1Δ::LYS2* P_{HOP1}*-GFP-PCH2/pch2Δ::TRP1 dot1Δ::URA3*	This work
DP1744	BR1919-2N P_{HOP1}*-GFP-PCH2/pch2Δ::TRP1 nup2Δ::hphMX4*	This work
DP1745	BR1919-2N *zip1Δ::LEU2* P_{HOP1}*-GFP-PCH2/pch2Δ::TRP1 nup2Δ::hphMX4*	This work
DP1746	BR1919-2N *zip1Δ::LEU2* P_{HOP1}*-GFP-PCH2/pch2Δ::TRP1 orc1-3mAID::hphNT* P_{HOP1}*-OsTIR1::URA3 dot1Δ::kanMX6*	This work

DP1747	DP421 *zip1Δ::LYS2* P_{HOP1}*-GFP-NES-PCH2/pch2Δ::TRP1 dot1Δ::kanMX6*	This work
DP1768	DP421 P_{HOP1}*-GFP-NLS-PCH2 ndt80Δ::kanMX6*	This work
DP1769	DP421 *zip1Δ::LYS2* P_{HOP1}*-GFP-NLS-PCH2 ndt80Δ::kanMX6*	This work
DP1787	DP421 *spo11-3HA-6His::kanMX4 pch2Δ::TRP1*	This work
DP1789	DP421 *spo11-3HA-6His::kanMX4* P_{HOP1}*-GFP-PCH2/pch2Δ::TRP1*	This work
DP1791	DP421 *spo11-3HA-6His::kanMX4* P_{HOP1}*-GFP-NES-PCH2/pch2Δ::TRP1*	This work
DP1792	DP421 *spo11-3HA-6His::kanMX4* P_{HOP1}*-GFP-NLS-PCH2*	This work
DP1793	DP421 *spo11-3HA-6His::kanMX4* P_{HOP1}*-GFP-NLS-PCH2/pch2Δ::TRP1*	This work
DP1795	DP421 *PIL1-GBP-mCherry::hphMX6* P_{HOP1}*-GFP-NES-PCH2/pch2Δ::TRP1*	This work
DP1796	DP421 *zip1Δ::LYS2 PIL1-GBP-mCherry::hphMX6* P_{HOP1}*-GFP-NES-PCH2/pch2Δ::TRP1*	This work
DP1797	BR1919-2N *PIL1-GBP-mCherry::hphMX6* P_{HOP1}*-GFP-PCH2/pch2Δ::TRP1 LYS2/lys2*	This work
DP1802	DP421 *PIL1-GBP-mCherry::hphMX6 3HA-PCH2*	This work
DP1803	DP421 *zip1Δ::LEU2 PIL1-GBP-mCherry::hphMX6 3HA-PCH2*	This work
DP1811	DP421 *PIL1-GBP-mCherry::hphMX6* P_{HOP1}*-GFP-NES-PCH2/pch2Δ::TRP1 mek1Δ::natMX4*	This work
DP1812	DP421 *zip1Δ::LYS2 PIL1-GBP-mCherry::hphMX6* P_{HOP1}*-GFP-NES-PCH2/pch2Δ::TRP1 mek1Δ::natMX4*	This work
DP1813	BR1919-2N *PIL1-GBP-mCherry::hphMX6* P_{HOP1}*-GFP-PCH2/pch2Δ::TRP1 mek1Δ::natMX4 LYS2/lys2*	This work
DP1846	DP421 *PIL1-GBP-mCherry::hphMX6* P_{HOP1}*-GFP-NES-PCH2/pch2Δ::TRP1 spo11Δ::natMX4*	This work
DP1847	DP421 *zip1Δ::LYS2 PIL1-GBP-mCherry::hphMX6* P_{HOP1}*-GFP-NES-PCH2/pch2Δ::TRP1 spo11Δ::natMX4*	This work

*All strains are diploids isogenic to BR1919 and, unless specified, homozygous for the indicated markers. DP421 is a *lys2* version of the original BR1919-2N.
