# Supplementary material for: Pch2 orchestrates the meiotic recombination checkpoint from the cytoplasm
Source: PLoS Genet. 2021 Jul 14;17(7):e1009560. doi: 10.1371/journal.pgen.1009560 (PMC8312941; doi:10.1371/journal.pgen.1009560)
Supplement: S2 Table — (PDF) [file pgen.1009560.s010.pdf]

S2 Table. Plasmids

Plasmid name	Vector	Relevant parts	Source/Reference
pSS383	pFA6a	*GBP-mCherry::hphMX6*	A. Fernández-Álvarez
pSS393	pRS314	*TRIP1 CEN6* P_{HOP1}*-GFP-PCH2*	(Herruzo et al., 2019)
pSS408	pRS314	*TRIP1 CEN6* P_{HOP1}*-GFP-NES-PCH2*	This work
pSS421	pRS314	*TRIP1 CEN6* P_{HOP1}*-GFP-NLS-PCH2*	This work
pSK54	pRS306	*URA3 spo11-3HA-6His::kanMX4*	(Kee and Keeney, 2002)

Herruzo, E., B. Santos, R. Freire, J.A. Carballo, and P.A. San-Segundo. 2019. Characterization of Pch2 localization determinants reveals a nucleolar-independent role in the meiotic recombination checkpoint. *Chromosoma*. 128:297-316.

Kee, K., and S. Keeney. 2002. Functional interactions between SPO11 and REC102 during initiation of meiotic recombination in Saccharomyces cerevisiae. *Genetics*. 160:111-122.
