# Supplementary material for: Pch2 orchestrates the meiotic recombination checkpoint from the cytoplasm
Source: PLoS Genet. 2021 Jul 14;17(7):e1009560. doi: 10.1371/journal.pgen.1009560 (PMC8312941; doi:10.1371/journal.pgen.1009560)
Supplement: S3 Table — (PDF) [file pgen.1009560.s011.pdf]

S3 Table. Primary antibodies

Antibody	Host and type	Application* (Dilution)	Source / Reference
Hop1 (5C12E8)	Mouse monoclonal	WB (1:2000)	(Herruzo et al., 2019)
Hop1	Rabbit polyclonal	IF (1:300)	(Smith and Roeder, 1997)
Hop1-T318-ph	Rabbit polyclonal	WB (1:1000)	(Penedos et al., 2015)
H3-T11-ph	Rabbit polyclonal	WB (1:2000)	Abcam<br>ab5168
Pgk1 (22C5D8)	Mouse monoclonal	WB (1:5000)	Molecular Probes<br>459250
Pch2	Rabbit polyclonal	WB (1:2000)	(Herruzo et al., 2019)
Nsr1 (31C4)	Mouse monoclonal	IF (1:200)	ThermoFisher<br>MA1-10030
mAID (1E4)	Mouse monoclonal	WB (1:400)	MBL<br>M214-3
GFP (JL-8)	Mouse monoclonal	IF (1:200)	Clontech<br>632381

*WB, western blot; IF, immunofluorescence

Herruzo, E., B. Santos, R. Freire, J.A. Carballo, and P.A. San-Segundo. 2019. Characterization of Pch2 localization determinants reveals a nucleolar-independent role in the meiotic recombination checkpoint. *Chromosoma*. 128:297-316.

Penedos, A., A.L. Johnson, E. Strong, A.S. Goldman, J.A. Carballo, and R.S. Cha. 2015. Essential and Checkpoint Functions of Budding Yeast ATM and ATR during Meiotic Prophase Are Facilitated by Differential Phosphorylation of a Meiotic Adaptor Protein, Hop1. *PLoS One*. 10:e0134297.

Smith, A.V., and G.S. Roeder. 1997. The yeast Red1 protein localizes to the cores of meiotic chromosomes. *J Cell Biol*. 136:957-967.
