# Supplementary material for: Pch2 orchestrates the meiotic recombination checkpoint from the cytoplasm
Source: PLoS Genet. 2021 Jul 14;17(7):e1009560. doi: 10.1371/journal.pgen.1009560 (PMC8312941; doi:10.1371/journal.pgen.1009560)
Supplement: Attachment — Submitted filename: Response to reviewers PGENETICS-D-21-00557.pdf [file pgen.1009560.s014.pdf]

Response to comments from Editor and Reviewers.

We thank the editor and all reviewers for their constructive comments and suggestions.

In the responses below, lines refer to the "clean" version of the manuscript without the track-changes option activated.

Response to Editor

1. The manuscript refers to heterozygous and homozygous pHOP1-GFP-PCH2 constructs, but it appears that most of the "heterozygous" constructs are in fact hemizygous (i.e. over a pch2Δ). This distinction is important because a true heterozygous construct will express both GFP-Pch2 and wild type Pch2, which I think is not what you mean. Please revise the nomenclature to reflect this; alternatively, please consider as an alternate "1 copy" and "2 copies". Regardless of which nomenclature you settle on, please devote a paragraph, or at least a few sentences, at the beginning, to clearly explaining the situation, and why hemizygous constructs were mainly used (I assume that it is because the HOP1 promoter is stronger than the PCH2 promoter, and so a hemizygous GFP-PCH2 construct produces about the same amount of protein as a homozygous wild-type PCH2 strain).

We apologize for the erroneous nomenclature and we thank you for the correction. We now use the term hemizygous for *GFP-PCH2/pch2Δ* strains throughout the paper. In addition, we have elaborated a bit more on the explanation for the use of the *HOP1* promoter to drive *GFP-PCH2* expression (lines 201-206). As we showed in our previous paper (Herruzo et al., Chromosoma 2019, 128:297-316), expression of *GFP-PCH2* from its own promoter produces very low levels of the protein, which are notably below normal endogenous Pch2 levels. The use of the stronger *HOP1* promoter, which, like *PCH2*, is also induced during prophase I, compensates for that deficiency and results in near-physiological protein levels. For the purpose of documenting this response, we attach a cropped version of Figure 4A of the Chromosome 2019 paper showing the relevant information (Figure I for Response Letter). In the current manuscript, we also show that hemizygous P_{HOP1}-*GFP-PCH2* strains produce protein levels quite comparable to those of wild-type *PCH2* strains (Fig 2A).

2. If my reading of the materials and methods is correct, then the nuclear/cytoplasmic GFP ratio is comparing GFP signal/unit area, not total GFP signal in nucleus versus cytoplasm. This is fine, as long as the ratio of nucleus/cytoplasm total area is relatively invariant. But have you considered looking at total nucleus GFP/total cytoplasm GFP ratio? I think that it would reveal that there is more Pch2 is in the cytoplasm than in the nucleus in wild-type cells. Regardless of which ratio you use, it would be useful to say somewhere the average ratio of nucleus/cytoplasm total area, so an interested reader could make the calculation themselves.

Yes, your reading is correct; we are representing the ratio of the "mean intensity" of nuclear/cytoplasmic fluorescence. In FIJI, this parameter is the average grey value within the selection; that is, the sum of values of all the pixels divided by the total number of pixels. Thus, it can be considered that this parameter represents the "concentration" of the fluorescence within the nucleus or the cytoplasm, and we plot the ratio of these values. Of course, it is possible, and that is indeed the case as you mention, that in the wild type there is more total GFP-Pch2

fluorescence in the cytoplasm, which is larger, but it is more concentrated in the nucleus/nucleolus, which is smaller.
In our measurements we also have the "Integrated Density" values representing total fluorescence in the area selected. However, we would like to point out that when making the selection of the nuclear and cytoplasmic areas using the own GFP fluorescence as indicator, it is not always possible to get a clear-cut definition, because of the non-uniform shape of the nucleus, the position of the irregular nucleolus towards one side of the nucleus, the variable size of the vacuole in the cytoplasm, etc. The use of this parameter (total fluorescence) for plotting the nuclear/cytoplasmic ratio, as you suggest, shows the same trend; please, see new S8 Fig.
Nevertheless, we consider that for illustrating how the balance between nuclear/nucleolar and cytoplasmic Pch2 varies among the different strains tested, which is our main purpose, it is best to maintain the ratio of the mean intensity as originally presented in the main Figures, because it is a more precise measurement for the concentration of Pch2 in each compartment. In any case, following your suggestion and also that of Reviewer#3, we include a supplementary figure showing the total nuclear signal/total cytoplasmic signal ratio (S8 Fig; lines 777-778), and we provide raw data for all the measurements (including area, mean grey value and integrated density) in an excel supplemental data file to make then available for the interested reader (S1 File).

3. lines 227-230. For the non-specialist reader, please say that Hop1-T318phos is being used as a measure of checkpoint activity

We have reinforced that notion (lines 234-236).

*4. Either in figures where t-test comparisons are made, or in the materials and methods, please indicate what p values an asterisk means (i.e. what is *,**,***, etc.)*

We have added that information in the materials and methods section (lines 800-801) and in the supplemental data file containing the Statistic summary (S2 File).

5. Please quantify western blots (using Pgk1 as a standard). I think it's too much to include the numerical data in figures, so including the values in a supplementary file (the same one as is used for reporting other underlying numerical data would work) would be fine.

As suggested, numerical raw data and the relativization to Pgk1 for western blots is provided in the Supplemental Data File (S1 File).

6. Reviewer #3 has suggested several additional experiments, all of which are nice ideas, but (as the reviewer says in their comments to the editor) none of which are in my opinion indispensable. I also think that, given the high levels of proteases in meiotic yeast cells, some of the suggested approaches (subcellular fractionation, co-IP) would be technically very challenging.

We thank the reviewer for the suggested experiments that will be definitely considered for future work. As you point out, at least in yeast, those experiments are quite challenging and would require a considerable amount of time/work to try and set up the conditions. With regards to the subcellular fractionation, I am not really convinced that it would really provide superior resolution than subcellular localization by fluorescence microscopy, although it could be certainly useful in the

future to determine where specific modifications/interactions occur. With regards to the interaction between Hop1 and Pch2, *in vitro* studies have shown that it is very transient (Chen et al., PNAS 2014), and it would require the use of an ATP hydrolysis deficient version of Pch2 and a significant amount of time working out the experimental conditions to be detected (in case it is possible…).

*7. Reviewer #2 commented on the references section, which I agree needs proofreading to fix all of the formatting inconsistencies that your favorite reference manager creates. I think that reviewer #2 meant that only proper names and the first word of the title should be *capitalized* (not italicized). Gene and species names, of course, should be italicized.*

We apologize for the inconsistencies created by Endnote; we have now manually corrected each reference in the revised version of the manuscript.

Response to Reviewer#1

1. The model of Pch2-mediated "unlocking" of Hop1 to mediate its nuclear import and Red1 association is compelling. However, Pch2 is not required for this process in an otherwise-normal meiosis: pch2-delta cells proceed through meiosis and have high spore viability, indicating that sufficient Hop1 and Red1 localize to chromosomes to support DSB formation, impose homolog bias through Hop1 phosphorylation and Mek1 activation, etc. Thus, Hop1 must have an inherent locked-unlocked equilibrium that enables limited recruitment to chromosomes without Pch2. I think it is important that the authors address this point alongside their discussion of Pch2's role in situations where recombination or synapsis is compromised.

We have included a paragraph in the discussion along the lines suggested by the reviewer (lines 672-677).

2. Can the authors briefly address why they used the HOP1 promoter to drive expression of their various PCH2 alleles?

Please, see the response to Editor's point 1

3. I'm not sure the experiments in Figure S2 are necessary. These plasmid-based constructs are quickly (and correctly) substituted for GFP-fusion constructs integrated at the PCH2 locus. What does the inclusion of these strains/experiments add to the paper?

We agree with the reviewer that this Fig S2 does not provide substantial additional information. However, we consider that this Figure is very helpful for a fluent and ordered narrative flow. We first illustrate in Fig S2, using plasmid constructs transformed into *zip1Δ* strains, that the addition of a NES/NLS is a valuable tool to redirect GFP-Pch2 towards the cytoplasm/nucleus. Once this is established, then we generate the whole set of "integrated" hemizygous and homozygous strains in both *ZIP1* and *zip1Δ* background, analyze protein levels (Fig 2A), determine sporulation efficiency of all versions (Fig 2B and 2C) and, based on all that information, we choose the set of strains (all the hemizygous and also the homozygous *GFP-NLS-PCH2*) for further in-depth cytological analyses in Figs 3 and 4. Thus, we prefer to maintain S2 Fig as the initial support for the development of the set of all integrated strains.

4. I would recommend moving Figure 3B to supplemental information or removing it entirely. To me it's confusing to have essentially the same information presented in Figure 3A and 3B.

We understand the reviewer's confusion. The main and only purpose of the previous Fig 3B is to show fields containing more cells so that the reader can get an visual idea (besides the quantification) of the cell-to-cell variability and the representativeness of the individual cells shown in Fig 3A, especially for cases such as *GFP-NES-PCH2* in which the residual nucleolar signal can vary. Thus, according with the reviewer's comment, we have decided to move the old Figure 3B to the supplemental information (now S3 Fig) so an interested reader can still have access to it. Also, and in line with the next reviewer's comment, we have decided to make a single call to this S3 Fig at the beginning of the section (line 277) to avoid overwhelming the reader with the successive calls to the individual subpanels of this S3 Fig in addition to those of the main Fig 3A.

5. The panel labels in Figure 3A and 4A are somewhat confusing. I will leave it to the editor/journal to decide what kind of labeling changes to make.

We also concede that the labeling of the subpanels could be, I don't know if confusing, but at least arduous. However, we consider that it is absolutely necessary referring to each specific subpanel corresponding to each particular strain in the text; otherwise, without this direction, the reader may get lost. We were debating this issue among coauthors even before the first submission, and we came up with this "sub-labeling" using lowercase lettering (i.e., Fig. 4Aa, 4Ab…) as the "least bad" option. I think that removing the calls to the subpanels of the previous Figure 3B (now moved to S3 Fig) could help to mitigate this issue to some extent.

6. For the model figure, I would recommend using "C-Hop1" and "U-Hop1" instead of "Hop1" and "Hop1" as the two states. I understand the authors' hesitance to make this strong prediction, but it is in fact what they propose in the Discussion, and it fits with all known biochemical and genetic data on the proteins in question. Then Hop1 would become C-Hop1 once again (bound to Red1 instead of its own C-terminus) when bound to chromosomes. Also, in the Mad2 literature, the term "Mad2*" was used historically to refer to one of the conformational states. I can't find the exact references now, but it is probably best to avoid the asterisk in any case*.

As we propose in the discussion, it is likely that the initial conformational change imposed by the cytoplasmic Pch2 involves the transition from the closed to the unbuckled state referred to by the reviewer, but do not have *in vivo* evidence for that yet. In addition, it is also likely that other mechanisms (i.e., posttranslational modifications) may impinge on this process. Thus, with "Hop1*" we try to reflect a state of Hop1 that is poised or predisposed for its chromosome incorporation and phosphorylation at T318 in a broad sense, not exclusively indicating the conformational status. Therefore, as the reviewer understands, we prefer to stay on the safe side and maintain the model as it is. Nevertheless, we have elaborated a bit more along this line on the discussion (also following the comment from reviewer#2; lines 645-648), and we have also slightly modified the legend of Fig 8 (model) for further clarification (lines 1178-1180).

7. Just a note: After reading the passage on lines 654-663 summarizing the model, I raised my hands up and yelled "Yes!" - this is the best synthesis of the developing evidence and model that I have yet read.

We thank the reviewer for this supportive comment

Response to Reviewer#2

Line 89, It seems misleading to say that chromosome synapsis is occurring "in parallel" with recombination since recombination is required for SC formation.

We have rephrased that sentence (lines 90-91).

Line 93, need a reference for the statement the SC provides an "environment for properly regulated recombination".

A reference is provided (line 94).

Line 111 In zip1Δ (italicized), the...

Corrected, thank you.

Line 113, While it is true that Mek1 prevents Rad54-Rad51 complex formation, allowing these complexes to form is not sufficient to allow the high levels of IS repair observed in a mek1Δ. Therefore Mek1 must have other targets that promote IH bias and this statement oversimplifies the situation. It would be more correct to say ...it inhibits DSB repair by intersister recombination in part by ...."

Corrected as suggested (line 116).

Line 128: The statement that Hop1 is excluded from fully synapsed chromosomes is commonly made but has always perplexed me given the many published pictures of SCs containing alternating domains of Hop1 and Zip1 by San-Segundo, Börner and other authors. So Hop1 is clearly on synapsed chromosomes. An explanation of this conundrum would be helpful.

A sentence and a reference have been added that may help to clarify, at least in part, the conundrum referred to by the reviewer (lines 133-135).

Line 200, a reference should be provided for the HOP1 promoter. Out of curiosity, why didn't the authors use the PCH2 promoter since it appears that the HOP1 promoter expresses PCH2 to a higher level than is observed with endogenous Pch2?

Please, see the response to Editor's point 1. Also, we now provide a reference for the meiosis-specific expression of *HOP1* (line 202).

Line 259: the "Pch2 variants" are alleles of the gene so this should be italicized: PCH2 (italicized) variants

Changed

Line 269 genomically instead of genomic

Corrected, thank you.

Line 303, Consistent with...

Corrected, thank you.

Line 341 "...Hop1 axial binding.." is confusing, perhaps "...Hop1 binding to axial elements" or "...Hop1 binding to chromosome axes".

We have modified the sentence according to the reviewer's suggestion (line 347).

Line 386: need a reference for using H3-T11 as a proxy for Mek1 activity

We now provide a reference (line 391).

Line 407 and throughout: whenever the authors say the "absence of X", the experiments that were done were genetic and therefore it is the absence of the gene, not the protein, that should be indicated.

We have made the suggested changes throughout the paper

Line 637: The authors make the intriguing suggestion both that the Hop1 closure motif is "unbuckled" from Hop1 in the cytoplasm and that this conformational change might expose a cryptic NLS. In support of this idea, the C-terminus of Hop1 where the closure motif is located is rich in arginine and lysines (RKISVSKKTLKSNW) which they might want to mention.

We have added a sentence following the reviewer's suggestion (lines 645-647).

Line 686: Standard nomenclature is to indicate genes that have been replaced by a marker with "Δ", eg., zip1Δ::LEU2. The presence of a colon alone indicates an insertion. Please add "Δ" to all deletion alleles.

We have added the deletion symbol in the materials and methods section (lines 700-702) and the strains list (S1 Table).

References: Only proper names and the first word of the title should be italicized. Also all gene names and genus species names should be italicized (et, ref 9, 10, 28 and throughout).

We thank the reviewer for his/her careful reading. As indicated in the response to the Editor's point#7, we had an issue with our reference manager software, but all the formatting mistakes and inconsistencies have been manually corrected in the revised version of the manuscript.

Response to Reviewer#3

-Please define NES and NLS in the abstract.

We have made the suggested modification (line 44).

-To better characterize the efficiency of the strategy used to change the location of Pch2 within the different cell compartments, I would like the authors to show by Western Blot the presence of Pch2 in the cytoplasmic and the nuclear subcellular fractions, at least for the GFP-NES-PCH2 and the GFP-NLS-PCH2 strains.

Please, see the response to Editor's point#6

- One prediction of the authors' proposed model would be that Hop1 and Pch2 would interact in the cytoplasm. I wonder if the authors could test it by co-IPing Pch2 or Hop1 from the cytoplasmic fraction of the different yeast strains used or by other methods like in situ proximity ligation assays.

Please, see the response to Editor's point#6

-Please provide a WB showing the expression levels of the native Pch2 and the GFP-tagged Pch2 of homozygous and heterozygous strains containing GFP-NES-PCH2 and the GFP-NLS-PCH2.

The levels of native Pch2 compared to those of GFP-Pch2 in hemizygous or homozygous strains are shown in Fig. 2A. As requested by the Editor, we now provide quantitative data for all WBs, including this one, in the supplemental data (S1 File).
The levels of GFP-Pch2 compared to those of GFP-NES-Pch2 and GFP-NLS-Pch2 are shown and quantified in Fig. 5C, 5D.

-Fig. 2. Is the sporulation efficiency of Zip1Δ significantly different than the one from Zip1Δ GFP-NLS-PCH2 (heterozygous strain)? If not, please justify this result.

We now mark in Fig. 2B that the sporulation efficiency of the *zip1Δ GFP-PCH2* hemizygous is significantly different ($p<0.0001$) than the one of the *zip1Δ GFP-NLS-PCH2* hemizygous, which is the relevant comparison. Since apparently the reviewer questions about the *zip1Δ* "single mutant", please note that it is also significantly different from *zip1Δ GFP-NLS-PCH2* ($p=0.0016$).

-line 277. Please provide evidence that suggests the nuclear fraction of GFP-NES-PCH2 is present in the nucleolus. I guess the authors infer this from the following experiments performed on spreads, but it is not clear from the current wording of this section.

We have reworded that statement to be less categorical at this point in the manuscript (lines 284-285).

-*Fig. 3C. I think it would be good to provide the raw data for the GFP signal of the nucleus and the cytoplasm as a supplementary figure. It seems that Zip1 mutation causes a clear reduction in the ratio of nuclear/cytoplasmic GFP in all strains but GFP-NES-PCH2. Is this just because most of the GFP signal is already in the cytoplasm in wild-type strains?*

Please see the response to Editor's point#2. The use of total signal for representing the nuclear/cytoplasmic ratio (new S8 Fig) indicates that the interpretation of the reviewer is correct.

- I think it would be good to show the background level of green fluorescence for the in vivo measurement experiments displayed in Fig. 3C, similar to what is displayed in 4B for pch2Δ.

Unlike the absolute measurements presented in Fig. 4B, the current Fig. 3B (old Fig 3C) shows a ratio of the nuclear/cytoplasmic signal; therefore, the display of a background signal is not applicable. In any case, an average background level has been subtracted from every individual measurement prior to ratio calculation in Fig. 3B. Furthermore, we have previously presented a quantitative comparison of the fluorescence cellular distribution of an untagged control and a strain expressing P_{HOP1}-*GFP-PCH2* (Figure S4 from Herruzo et al., Chromosoma 2019). For the purpose of this response letter, a cropped version of the relevant panels from that published Figure is attached to this response (Figure II for Response Letter).

- Line 503. Does the constitutive activation of the checkpoint observed in zip1Δ GFP-NES-PCH2 PIL1-GBP-mCh depends on SPO11?

As suggested by the reviewer, we have tested the *SPO11* dependence. We now show that deletion of *SPO11* alleviates the tight sporulation block of both *GFP-NES-PCH2 PIL1-GBP-mCh* and *zip1Δ GFP-NES-PCH2 PIL1-GBP-mCh* strains, indicating that, indeed, the constitutive checkpoint activation does depend on *SPO11* (line 506). The results are presented in a new panel in S6D Fig. The new *spo11Δ* strains constructed have been added to the S1 Table.

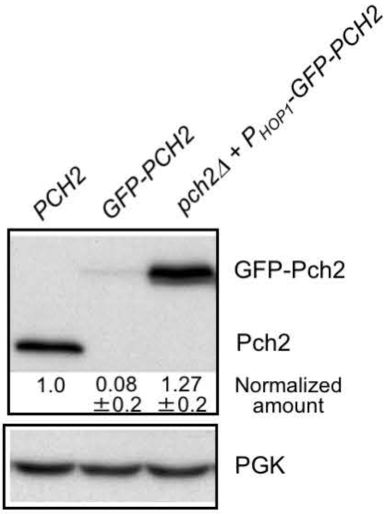

Figure I for Response Letter. Production of endogenous untagged Pch2 (lane 1), endogenous GFP-tagged Pch2 expressed from its own promoter (lane 2), and GFP-Pch2 expressed from the *HOP1* promoter in a centromeric plasmid (lane 3). The panels shown here depict the published relevant information cropped from Fig. 4a of Herruzo et al., Chromosoma 2019, only for the purpose of the response to Editor and Reviewers.

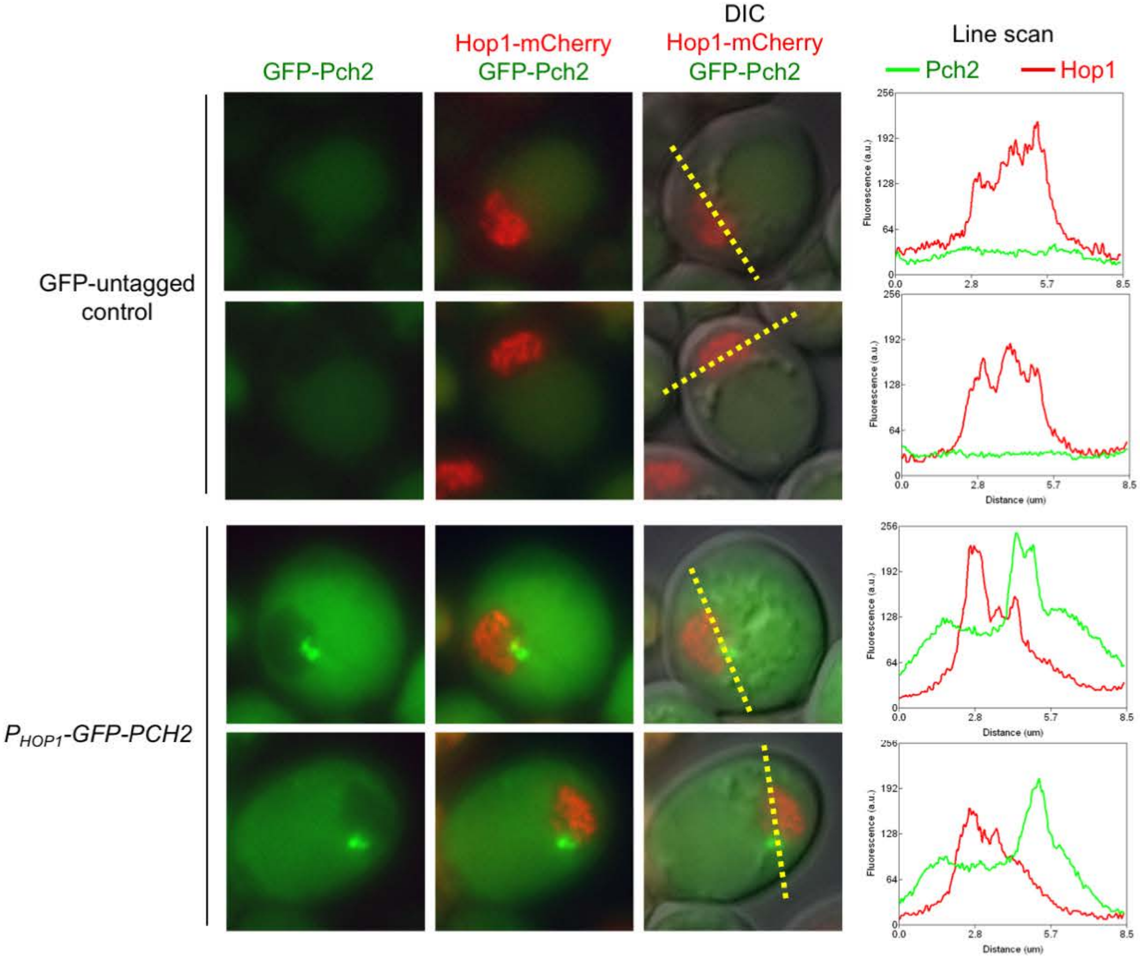

Figure II for Response Letter. Fluorescence microscopy images and the corresponding Line Scan plots of *zip1Δ* cells expressing either untagged *PCH2* or P_{HOP1}*-GFP-PCH2*, as indicated. All strains also express *HOP1-mCherry* in heterozygosis. The relevant panels shown here, only for the purpose of the response to Reviewer#3, are a cropped image of published Figure S4 in Herruzo et al. Chromosoma 2019.
